# Primary care physicians’ perceptions of practice improvement as a professional responsibility: a cross-sectional study

**DOI:** 10.1080/10872981.2018.1474700

**Published:** 2018-05-16

**Authors:** Christopher R. Stephenson, Christopher M. Wittich, Joel E. Pacyna, Matthew K. Wynia, Omar Hasan, Jon C. Tilburt

**Affiliations:** a Division of General Internal Medicine, Mayo Clinic, Rochester, MN, USA; b Biomedical Ethics Research Program, Mayo Clinic, Rochester, MN, USA; c Center for Bioethics and Humanities, Anschutz Medical Campus, University of Colorado, Aurora, CO, USA; d MaineHealth and Maine Medical Center, Portland, ME, USA; e Division of Health Care Policy and Research, Mayo Clinic, Rochester, MN, USA

**Keywords:** Continuing medical education, improvement in medical practice, Maintenance of Certification, professionalism, quality improvement

## Abstract

Continuous quality improvement is a component of professionalism. Maintenance of Certification (MOC) is a mechanism in the USA for physicians to keep current with medical knowledge and contribute to practice improvement. Little is known about primary care physicians’ perceptions of the practice improvement (Part IV) components of MOC. We aimed to determine primary care physicians’ perceptions of their professional responsibility to participate in Part IV MOC. This was a cross-sectional study of primary care physicians using the American Medical Association Masterfile. We developed a nine-item survey, designed from expert consensus and literature to determine views on Part IV MOC as a professional responsibility. We surveyed 1500 randomly selected primary care physicians via mail from November 2014 to May 2015. The response rate was 42% (627 of 1,500): 47% (273 of 585) were family practitioners and 49% (289 of 585) were internists. Factor analysis revealed a two-factor survey, with five items pertaining to positive views of MOC Part IV and four items pertaining to negative views. Internists were more likely to view MOC Part IV as time consuming (82.0% vs. 70.3%, *P* = .001), expensive (50.9% vs. 38.8%, *P* = .004), and not relevant to practice (39.1% vs. 23.8%, *P* < .001). Family medicine practitioners were more likely to view MOC Part IV as improving patient care (64.5% vs. 48.8%, *P* < .001) and maintaining professional responsibility (48.7% vs. 32.5%, *P* < .001). Regardless of specialty, most physicians viewed MOC Part IV as time intensive, not beneficial for career advancement, and not a professional responsibility. Family medicine practitioners demonstrated more positive views of MOC Part IV. The difference between family medicine practitioners and internists could be related to the ABIM MOC controversy. Future changes to practice improvement requirements could focus on limiting time requirements and on clinical relevance.

**Abbreviations**: ABIM: American Board of Internal Medicine; AMA: American Medical Association; CQI: continuous quality improvement; IRB: institutional review board; MOC: Maintenance of Certification; QI: quality improvement

## Background

Maintaining a commitment to lifelong learning, including continuous quality improvement (CQI) in health care, is a core component of medical professionalism [1,2]. Traditionally, board certification has been the primary method for physicians to demonstrate their competence; recently, with the creation of the American Board of Internal Medicine (ABIM) Maintenance of Certification (MOC) Part IV activities, it has been used to demonstrate commitment to quality improvement (QI) [3–5]. Although board certification has some proven value, the value of MOC Part IV activities is less clear [6–10].

Physicians may accept or even support MOC’s general framework, but the implementation of MOC has led to dissatisfaction, with physicians citing particular concerns over cost and excessive time requirements [11–15]. Almost 70% of general internists are dissatisfied with MOC, and internists are less likely to maintain certification than other specialists [16]. Furthermore, current implementation for MOC is costly, with limited data on its relevance for clinical practice [17–21]. Despite this dissatisfaction, evidence suggests that MOC can improve some clinical measures, such as self-reported knowledge, communication with patients, and clinical care [22–25]. Previous studies have assessed physicians’ views on MOC in general, but less is known about the specific issue of requiring CQI work as part of MOC.

Many physicians already engage in CQI, and previous studies have shown promising outcomes for CQI: Physician engagement in practice improvement modules has led to behavioral changes and self-reported practice improvements [26,27]. Furthermore, evidence suggests that meaningful reflection leads to increased success with CQI projects [28]. However, despite the importance of CQI, little is known about how internal medicine physicians view their professional responsibility to participate in the CQI component of MOC compared with physicians in other specialties, particularly other primary care providers.

To fill this gap, as part of a national survey of primary care physicians on QI, we examined their views toward requiring QI to maintain board certification. We gathered self-reported experiences and impressions of the MOC Part IV Practice Assessment, including (1) self-reported perceptions of the Part IV Practice Assessment component of MOC as a professional responsibility and (2) personal and practice characteristics of physicians associated with agreeing with such a responsibility.

## Methods

### Study data and methods

Data were gathered from a mailed physician survey entitled ‘Physicians’ Experiences With QI in the Real World,’ which was developed through standard survey methods (including gathering of existing measures and cognitively testing new measures) and conducted in four waves from November 2014 through May 2015. The initial survey sample comprised 1,500 primary care physicians in the American Medical Association (AMA) Physician Masterfile, which includes nearly all physicians licensed to practice in the USA. Eligible physicians were those involved in direct patient care from three primary care specialties: internal medicine, family medicine, and general practice. Physician specialty was initially assigned according to the Physician Masterfile data and was verified in the survey. Within this sample, 1,000 were randomly selected, and the remaining 500 were oversampled from among Hispanic or Latino (*n* = 250) or African-American (*n* = 250) physicians.

The survey was distributed by the Survey Research Center at Mayo Clinic in Rochester, Minnesota. The first wave contained a $10 cash incentive to discourage gatekeeper filtering and encourage physician participation. The second and third waves were mailed to nonresponders at 27 and 60 days after the initial mailing. The third survey mailing to nonresponders contained a branded pen as a token incentive, and responders were mailed the token incentive retroactively to equalize the incentive across our sample. After the third mailing, the AMA research staff sent a facsimile reminder to nonresponders. Finally, the AMA staff conducted reminder telephone calls to nonresponders with an opportunity for physicians to reply to a limited set of survey questions by phone.

Since some components of our survey fielding were conducted at Mayo Clinic and other components were conducted by AMA research staff, both the Mayo Clinic Institutional Review Board (IRB) and the AMA IRB reviewed the study protocol and deemed the study exempt.

### Survey instrument

Survey items were designed from expert opinion, iterative group consensus, and existing literature. Our survey instrument contained several items derived from other physician surveys that explored health information technology adoption, QI, and health disparities [29]. For the present study, we used a set of survey items to assess physician participation in the MOC Part IV Practice Assessment and their views about it. Items addressed whether the physician planned to perform MOC activities, including Part IV activities, and whether the physician agreed or disagreed with several positive and negative statements about MOC Part IV activities. A total of nine items were developed, cognitively tested, and included in the final instrument.

### Data management and analysis

Survey responses were double-entered, cleaned, and checked for quality by trained staff who followed standardized procedures. Frequency (percentage) or mean (SD) were calculated for demographic variables, as appropriate. Responses to questions about physician-reported enrollment in MOC Part IV QI work, value, feasibility, and appropriateness of doing an MOC Part IV QI project were also summarized. All analyses were performed with SAS version 9.4 software (SAS Institute Inc., Cary, North Carolina). Data weights for responders were generated with an iterative ranking method to account for race/ethnicity, sex, and age group, so that the results would be nationally representative. Population distribution information for the weightings was obtained from the *Physician Characteristics and Distribution in the US, 2014* [30].

### MOC attitudinal variable characteristics

Factor analysis was used on the items exploring views about MOC Part IV to identify possible subdomains among the nine statements (a 5-point agreement scale was used after the response ‘don’t know’ was reclassified as ‘neutral’). Survey items with factor loadings of 0.50 or more were identified and grouped according to the factors to which they loaded most highly. Items about institutional support for MOC and institutional requirements to carry out MOC activities were conceptually distinct and were removed from the factor analysis. Remaining items were assigned to two subscales: one was a positive subscale with five items that posited that Part IV activities could improve practice, advance careers, promote professionalism, be a professional responsibility, and be intrinsically fun or interesting. The second subscale had four items that posited that Part IV activities were a negative experience because of their irrelevance, hassle, expense, and redundancy. The Cronbach α was 0.838 for questions associated with positive attitudes and 0.684 for questions associated with negative attitudes. For these 2 domains we created composite scores comprising the physician’s average response across the items in each domain. The composite scores had a possible range of 1 to 5, with 5 representing the most extreme attitude on the spectrum. We used these composite scores to examine associations between physician characteristics and positive or negative attitudes toward MOC Part IV activities in a set of unadjusted linear regression models, followed by a single multivariable regression model for each score including all characteristics. *P*-values of .05 or less were considered significant.

## Results

### Study participants

A total of 1,500 surveys were distributed, and 627 surveys were returned (response rate, 42%). A little over half the participants, 58% (344 of 598), had been in practice for 10 years or less, with the remainder, 42% (254), in practice for 11 years or more (Table 1). Of the 585 physicians who reported their specialty, 289 (49%) were internists, 273 (47%) were family practitioners, and 23 (4%) were in general practice or other type of practice. Sixty percent of participants (379) were men, 34% (212) were older than 55 years, and 95% (576) were board certified.

**Table 1.** Characteristics of 627 US primary care physicians^a.^

Characteristic	No. (%)
Male	379 (60.4)
Age >55 years	212 (33.8)
Board certified	576 (91.9)
Years in practice (*n* = 598)	
<1	29 (4.8)
1–5	196 (32.8)
6–10	119 (19.9)
≥11	254 (42.5)
Region^a^	
Northeast	123 (19.6)
Midwest	109 (17.4)
South	239 (38.1)
West	149 (23.8)
Puerto Rico	7 (1.1)
Specialty (*n* = 585)	
Family medicine	273 (46.7)
Internal medicine	289 (49.4)
General practice	16 (2.7)
Other	7 (1.2)
Practice type^b^	
Solo	121 (19.3)
Single-specialty group	107 (17.1)
Multispecialty group	101 (16.1)
Community clinic or public clinic	42 (6.7)
Hospital-owned office (employee)	93 (14.8)
Hospital inpatient (employee)	72 (11.5)
Staff model HMO	23 (3.7)
Other	79 (12.6)

HMO: health maintenance organization.

^a^Unless indicated otherwise, the number of respondents was 627.

^b^Total is 638 because some physicians reported more than 1 practice type.

### Survey results

Among all 627 respondents, 62% were enrolled in MOC, 22% were not enrolled but planned to enroll, and 16% did not plan to enroll (Table 2).

**Table 2.** Self-reported experiences and impressions of MOC Part IV among 627 US primary care physicians.

Characteristic or survey item	No. (%)^a^
MOC participation (*n* = 551)	
Enrolled in MOC	341 (61.9)
Not enrolled but plan to enroll	121 (22.0)
Do not plan to enroll	89 (16.2)
Agree ‘Doing a MOC Part IV QI project …’	
‘takes too much time’	472 (75.3)
‘can help improve quality of care’	346 (55.2)
‘supports professionalism’	304 (48.5)
‘is too expensive’	281 (44.8)
‘is redundant with my other QI work’	277 (44.2)
‘is a professional responsibility’	250 (39.9)
‘is/would be interesting/fun to do’	205 (32.7)
‘is not relevant to my practice’	198 (31.6)
‘is helpful for career advancement’	136 (21.7)

MOC: Maintenance of Certification; QI, quality improvement.

^a^Unless indicated otherwise, percentages are based on 627 respondents.

Physicians’ views about MOC Part IV varied: 22% agreed that Part IV activities are helpful for career advancement, 55% agreed that the activities can improve quality of care, and 40% agreed that carrying out the activities is ‘a professional responsibility,’ but most agreed that the activities take too much time (75%), are too expensive (45%), and are not relevant to practice (32%) (Table 2).

### Attitude and perception scores

The mean (SD) score for the positive items taken as a group was 2.8 (1.0) out of 5 points on a Likert scale; the mean (SD) score for the negative items was 3.3 (0.9). Physicians were more likely to hold positive views if they were not board certified (3.3 vs. 2.8, *P *= .01) and family medicine physicians (3.0 vs. 2.7, *P *= .002); these differences remained significant after adjusting for all physician characteristics simultaneously. Mean negative attitude scores were higher for internal medicine physicians (3.4 vs. 3.1, *P *< .001), a finding that remained after adjustment for all other physician characteristics. Male physicians had more negative attitudes than female physicians (mean, 3.3 vs. 3.2), but only the adjusted *P*-value was significant (unadjusted *P *= .07, adjusted *P *= .05) (Table 3).

**Table 3.** Factor analysis of survey respondents with positive and negative attitude survey scores.

		Positive attitude score			Negative attitude score		
Characteristic	*N*	Mean (SD)	*P*^a^	*P*^b^	Mean (SD)	*P*^a^	*P*^b^
Sex			.19	.16		.07	.05
Female	226	2.90 (1.00)			3.21 (0.88)		
Male	352	2.79 (0.95)			3.34 (0.84)		
Age, years			.96	.69		.43	>.99
≤55	383	2.84 (0.97)			3.27 (0.83)		
>55	195	2.83 (0.96)			3.33 (0.91)		
Board certified			.01	.008		.36	.21
No	28	3.28 (0.79)			3.14 (0.61)		
Yes	546	2.81 (0.97)			3.29 (0.87)		
Years in practice			.20	.20		.97	.85
0–5	206	2.93 (0.96)			3.28 (0.81)		
6–10	110	2.78 (0.94)			3.30 (0.80)		
≥11	238	2.79 (0.98)			3.29 (0.92)		
Region			.62	.75		.11	.35
Northeast	113	2.83 (0.91)			3.40 (0.80)		
Midwest	105	2.74 (1.00)			3.39 (0.90)		
South	215	2.88 (0.95)			3.19 (0.85)		
West	138	2.79 (1.00)			3.31 (0.87)		
Specialty			.002	.01		<.00	.001
IM, GP, or other	289	2.71 (0.98)			3.44 (0.89)		
Family medicine	253	2.97 (0.93)			3.11 (0.79)		

GP: general practice; IM: internal medicine.

^a^Unadjusted *P*-value.

^b^Adjusted *P*-value from model, including all variables listed in the table.

When individual items were considered, internists were more likely to view MOC Part IV as too time consuming (82% vs. 70%, *P *= .001), too expensive (51% vs. 39%, *P *= .004), and not relevant to practice (39% vs. 24%, *P *< .001). Family medicine practitioners were more likely to view MOC Part IV as improving patient care (65% vs. 49%, *P *< .001) and maintaining professional responsibility (49% vs. 32%, *P *< .001). Compared with internists, family medicine practitioners were also more likely to be enrolled in MOC (71% vs. 54%, *P *< .001) (Table 4).

**Table 4.** Self-reported experiences and impressions of MOC Part IV among internal medicine and family medicine physicians.

	Internal medicine (*n* = 266)	Family medicine (*n* = 244)	
Characteristic or survey item	No. (%)	No. (%)	*P*-Value^a^
MOC participation			
Enrolled in MOC	143 (53.8)	172 (70.5)	<.001
Not enrolled but plan to enroll	63 (23.7)	46 (18.9)	
Do not plan to enroll	60 (22.6)	26 (10.7)	
Agree ‘Doing a MOC Part IV QI project …’			
‘takes too much time’	237 (82.0)	192 (70.3)	.001
‘can help improve quality of care’	141 (48.8)	176 (64.5)	<.001
‘supports professionalism’	133 (46.0)	141 (51.6)	0.18
‘is too expensive’	147 (50.9)	106 (38.8)	0.004
‘is redundant with my other QI work’	130 (45.0)	124 (45.4)	0.92
‘is a professional responsibility’	94 (32.5)	133 (48.7)	<.001
‘is/would be interesting/fun to do’	87 (30.1)	100 (36.6)	0.10
‘is not relevant to my practice’	113 (39.1)	65 (23.8)	<.001
‘is helpful for career advancement’	63 (21.8)	61 (22.3)	0.88

MOC: Maintenance of Certification; QI: quality improvement.

^a^χ^2^ test.

## Discussion

The recent debates regarding MOC, particularly Part IV, led to the retraction of the ABIM’s MOC Practice Assessment requirement for internal medicine. However, Practice Assessment is a requirement for other primary care specialties, including family medicine. These data suggest that professional opinion is divided on the role of MOC Part IV. Taken together, most respondents agreed that MOC Part IV can improve quality of care. However, the degree of agreement with this, and with other related attitudinal measures, varied sharply by professional specialty.

Overall, most sampled physicians, regardless of specialty, viewed MOC Part IV (as of late 2014 and early 2015) as a time-intensive process that was not beneficial for career advancement and was not a professional responsibility. Internists were even less likely than family medicine physicians to view Part IV as a professional responsibility. Interestingly, even though many physicians did not view Part IV as a professional responsibility, they did feel that it was relevant to their practice and could improve patient care. In the increasingly busy schedules of primary care physicians, the time-intensive nature of Part IV may contribute to these negative attitudes. Physician concerns for time requirements were the most common negative perception. It is unknown whether decreasing the time involved to complete Part IV requirements would improve respondents’ perceptions of Part IV, but it is remarkable that a majority of respondents could identify the value of Part IV for improving the quality of patient care, despite its operational hassle. One could speculate that future improvement in the operational dimensions of Part IV, including reducing the time requirements, may positively influence general attitudes about the value of MOC.

Differences between internists and family medicine physicians toward MOC Part IV were notable. Family medicine physicians were more likely to have higher positive attitude scores toward MOC Part IV, while internists were more likely to have higher negative attitude scores. Interestingly, internists were significantly less likely to be enrolled in MOC compared to family medicine colleagues. Since internists also had more negative attitudes toward MOC, and since fewer were enrolled in MOC, internists may have preconceived negative biases toward MOC that reflect the heated controversy in late 2014 [7].

Although the ABIM called for the retraction of Part IV until 2018, our data suggest that internists do see value in Practice Assessment. Rather than completely removing Part IV, the ABIM could focus on making operational elements of Part IV more flexible and relevant to the daily practice of internal medicine. Family medicine may provide important models for implementing such requirements in a manner that fits with clinical practice.

Ensuring a long-term commitment to practice improvement as a core value of medical professionalism must be reinforced throughout medical training [31]. Emphasizing the professional responsibility of practice performance assessment in undergraduate and graduate medical training, and providing mentorship with concrete examples of its value, may be crucial for sustaining that commitment.

### Strengths and limitations

Even though we used a comprehensive and representative database for physicians, the AMA Physician Masterfile, our response rate was predictably modest [32]. The timing of our survey was both a strength and a potential weakness. The 2014 MOC controversy may have preferentially primed respondents, particularly internists, to have extreme opinions, and MOC media coverage may have influenced our survey results. Further study comparing perceptions after the February 2015 ABIM MOC retraction could shed light on the durability of the opinions we documented. Strengths of the study include use of a national database with a representative population and use of a dedicated survey research center.

## Conclusions

While most primary care physicians reported that MOC Part IV improves care, family medicine practitioners were more likely than internists to view MOC Part IV as a professional responsibility and as a valuable activity. Future changes to practice improvement requirements could focus on operational modifications, such as limiting time requirements and focusing on clinical relevance. By improving the operational hurdles of MOC Part IV, physicians can focus on the broader, lifelong learning goal of practice performance assessment and not feel burdened by the operational hassles and frustrations of simply trying to meet these MOC requirements.

## Declarations

***Ethics Approval and Consent to Participate***

The Mayo Clinic IRB and the AMA IRB reviewed the study protocol and deemed the study exempt. All respondents agreed and consented to participate.

***Consent for Publication***

Not applicable.

***Availability of Data and Material***

The datasets used and/or analyzed during the current study are available from the corresponding author on reasonable request.
